# An Information-Theoretic Bound on *p*-Values for Detecting Communities Shared between Weighted Labeled Graphs

**DOI:** 10.3390/e24101329

**Published:** 2022-09-21

**Authors:** Predrag Obradovic, Vladimir Kovačević, Xiqi Li, Aleksandar Milosavljevic

**Affiliations:** 1 School of Electrical Engineering, University of Belgrade, 11000 Belgrade, Serbia; 2 Department of Molecular and Human Genetics, Baylor College of Medicine, Houston, TX 77030, USA; 3 Quantitative and Computational Biosciences Program, Baylor College of Medicine, Houston, TX 77030, USA

**Keywords:** pattern discovery, connectedness, information theory, community detection

## Abstract

Extraction of subsets of highly connected nodes (“communities” or modules) is a standard step in the analysis of complex social and biological networks. We here consider the problem of finding a relatively small set of nodes in two labeled weighted graphs that is highly connected in both. While many scoring functions and algorithms tackle the problem, the typically high computational cost of permutation testing required to establish the *p*-value for the observed pattern presents a major practical obstacle. To address this problem, we here extend the recently proposed CTD (“Connect the Dots”) approach to establish information-theoretic upper bounds on the *p*-values and lower bounds on the size and connectedness of communities that are detectable. This is an innovation on the applicability of CTD, broadening its use to pairs of graphs.

## 1. Introduction

Numerous social and biological networks can be modeled as graphs where each node is uniquely labeled and where edge weights represent strength of connections, such as strength of connection of two individuals within a social network or correlations between two genes or two metabolites in a biological network. Extraction of subsets of highly connected nodes (“communities” or modules) is a standard network analysis step. A wide array of community detection algorithms exist [1], some of them based on information theory [2,3]. We here consider the related problem of finding a relatively small set of nodes in two labeled weighted graphs that is highly connected in both. Notably, the nodes may be connected using only partially overlapping or even completely non-overlapping sets of edges within the two graphs. Moreover, edge weights are taken into account. While many scoring functions and algorithms may tackle the problem, the computational cost of permutation testing that is required to establish the *p*-value for the observed pattern of high connectedness of the corresponding nodes in the two graphs presents a major obstacle in practical applications to large networks. To address this problem, we here extend the recently proposed CTD (“Connect the Dots”) information-theoretic approach [4].

More formally, given two weighted undirected graphs $G_{1}$ and $G_{2}$ of same size with unique node labels, the set of labels being identical for the two graphs, consider the problem of detecting a subset *S* of node labels where corresponding nodes are highly connected in both graphs. Specifically, we focus on establishing information-theoretic upper bounds on the *p*-values and lower bounds on the size and connectedness of communities that are detectable. Our results are independent of the algorithm used to detect *S* and thus pave the way to many practical implementations. For example, the method can be used to test the statistical significance of shared network substructures provided by some “external user”, no matter the context of data represented by the networks or the procedure used to choose the observed substructures which correspond to *S*. If the chosen substructures contain a shared connectivity pattern, it will be detected and its statistical significance will be measured.

The problem of discovering a shared highly connected node module has broad applications in biology. Namely, gene co-expression networks and metabolic networks are weighted undirected labeled graphs in which patterns of connectedness are very important to detect and evaluate. Our previous work illustrates successful application of information-theoretic methods to both gene expression networks in breast cancer [4] as well as to metabolic networks that model metabolomic perturbations in human inborn errors of metabolism [5,6]. A vast array of applications can be envisioned in social network analysis. For example, groups of individuals that form tightly-knit communities on several different social media platforms could be identified. The problem of finding a significantly connected common set of nodes is at some level related to the classical network theory problem of finding a maximum common subgraph (MCS). Despite being NP-hard, MCS remains important for its applications in chemoinformatics [7], protein function prediction [8], etc. However, the MCS problem is distinct as it is defined on graphs with unlabeled nodes and centers on finding the correspondence of nodes and edges as it seeks the largest common subgraph. In contrast, we are interested in nodes that may possibly be connected using distinct sets of edges in the two graphs. Moreover, unlike in MCS, in our case, the edge weights also count.

Our solution extends the Connect the Dots (CTD) [4] approach based on information theory that can be used in its current implementation to find a significantly connected sub-set of nodes within a given set *S* of nodes in the input weighted graph *G*. The method provides an upper bound on the *p*-value that measures how significant this connectedness is in *G*. CTD also finds a subgraph F whose nodes originate from *S*, which expresses the pattern of high connectedness that is measured by the *p*-value. It has been successfully employed in clinical diagnosis of 16 inborn errors of metabolism [4] as well as evaluation of other metabolic disorders [6]. CTD-based metrics outperforms rule-based biomarker models and shows comparable accuracy to pathway-based models, thus providing a valuable method for automated, quantitative and scalable diagnosis of metabolic diseases, especially those lacking clear pathway knowledge [4].

The main advantage of CTD over other candidate algorithms for connectedness discovery is its ability to calculate a *p*-value via use of information theory, without the need to conduct costly permutation testing. Next, a very powerful aspect of CTD is that knowledge about the whole graph *G* is not needed, just the information about its size and knowledge about the nodes in *S* and close to it (one or two hops from a node from *S*). These facts make it quick and efficient even for big graphs and ideal for the application of finding shared highly connected node modules.

**Figure 1:**
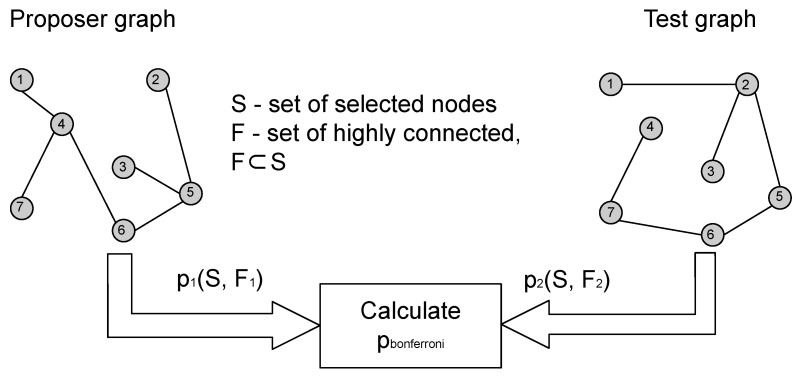
Process of calculating relation between the proposer and tester graphs.

The core idea of this paper is to use one of the input graphs as a *proposer* graph, while the other graph takes the role of a *tester* as shown in Figure 1. The *proposer* proposes a node subset *S*, and we use CTD to calculate the *p*-value for *S* in the *tester*. However, this *p*-value needs to be corrected for multiple testing and we do this by applying weighted Bonferroni correction. Weights for the weighted Bonferroni correction are chosen using Kraft–McMillan inequality [9,10] to construct a probability distribution on the power set of the proposer graph, based on the CTD encoding scheme and the algorithmic significance theorem [11]. Afterwards, the probability of *S* occurring in *G*_1_ according to the calculated probability distribution is used as the weight for weighted Bonferroni correction. The described procedure is an information theoretic algorithm based on CTD that can evaluate the statistical significance of connectedness of a node module in a graph pair, that is, solve the problem of shared highly connected node module detection.

## 2. Materials and Methods

In this section, we give a short review of the main features of the CTD algorithm and the mathematical apparatus applied in our work. Furthermore, we describe the methodology of conducting synthetic graph generation for the purpose of testing of our approach and list the set of software tools and platforms used in implementation.

### 2.1. Probability Distribution on the Power Set of G

Let G(V,E) be a weighted undirected graph and let P(V) denote its power set. As discussed in [4], running the CTD algorithm on *G* with the chosen node subset *S* yields an optimal bitstring encoding for *S*, constructed via the CTD’s encoding scheme. Let l(A) denote the length of the optimal encoding of the node subset *A*. The encoding scheme used by CTD satisfies the requirements for applying the Kraft–McMillan inequality to the set of encodings of P(V). Emulating the proof of Algorithmic significance theorem [11], the following inequality holds:

(1) $$\sum_{A\in P(V)}2^{-l(A)}\leq 1$$

Therefore, for some k<1, we have $\sum_{A\in P(V)}2^{-l(A)}=k$. Then, after dividing both sides with *k*, we obtain:

(2) $$\sum_{A\in P(V)}\frac{2^{-l(A)}}{k}=1$$

This generates a discrete probability distribution on the power set of *G*, where $P\left(A\right)=\frac{2^{-l(A)}}{k}$. Therefore, the probability of some node subset A∈P(V) and the pattern of its induced subgraph occurring in *G*, according to the probability distribution generated by the CTD’s encoding scheme, are given by:

(3) $$P\left(A\right)=\frac{2^{-l(A)}}{k}\geq 2^{-l(A)}$$

Note that, in order to get a bound on probability of a node subset *A*, we only need to run CTD to encode *A*, without the need to encode all possible subgraphs of *G*.

### 2.2. Applying Weighted Bonferroni Correction

Let $G_{1}\left(V_{1},E_{1}\right)$ and $G_{2}\left(V_{2},E_{2}\right)$ be weighted graphs with identical node labels ($V_{1}\equiv V_{2}$) and let *P* be a discrete probability distribution on the power set of $G_{1}$ generated by running the CTD encoding scheme on $G_{1}$, as discussed in the previous subsection. Alternatively, $G_{1}$ and $G_{2}$ can be graphs with an established node correspondence.

Let *S* be a subset of nodes of $G_{1}$ that was deemed significant in $G_{1}$. Let $p(S,G_{2})$ be a *p*-value of *S* in $G_{2}$, as calculated by CTD. Then, in order to acquire a *p*-value for the significance of *S* as a common subset of $G_{1}$ and $G_{2}$, we need to apply a correction for multiple testing, as we are choosing *S* as a subset of $P(V_{1})$. As *P* is a discrete probability distribution on $P(V_{1})$, we can take weights for weighted Bonferroni correction as

(4) $$w_{A}=P\left(A\right),A\in P\left(V_{1}\right).$$

Then, the weighted Bonferroni corrected *p*-value $p_{Bonferroni}$ is calculated as

(5) $$p_{Bonferroni}\left(S,G_{1},G_{2}\right)=\frac{p(S,G_{2})}{P(S)}$$

As shown in [4], by the direct application of the Algorithmic significance theorem [11] on the CTD encoding scheme as the coding algorithm, the CTD calculated *p*-value can be bounded as follows:

(6) $$p\left(S,G_{2}\right)\leq 2^{-d_{score}},$$

where $d_{score}$ can be calculated as a difference between the lengths of encodings given by the null hypothesis and the alternate hypothesis. To reiterate, when *S* is much smaller than *G*, the encoding given by the null hypothesis encodes each node in *G* with log2(|V|) bits. The encoding according to the alternate hypothesis is based on the CTD encoding scheme. It firstly encodes one of the nodes in *S* using about log2(|V|) bits. Afterwards, a probability-diffusion-based network walker is used to encode other nodes in *S*, by visiting nodes in descending order of probability diffused to them. Some nodes from *S* are possibly not encoded in this compressed manner and need to be “hardcoded” with log2(|V|) bits.

Plugging in Equations (3) and (6) into Equation (5), we have

(7) $$p_{Bonferroni}\left(S,G_{1},G_{2}\right)\leq \frac{2^{-d_{score}}}{2^{-l(A)}}$$

Using the notation described in [4] and writing Equation (7) in terms of the encodings given by the alternate hypothesis and null hypothesis, we obtain

(8) $$p_{Bonferroni}\left(S,G_{1},G_{2}\right)\leq \frac{2^{-(I_{0}\left(G_{2}\right)-I_{A}\left(G_{2}\right))}}{2^{-I_{A}\left(G_{1}\right)}}$$

Therefore,

(9) $$p_{Bonferroni}\left(S,G_{1},G_{2}\right)\leq 2^{-(I_{0}\left(G_{2}\right)-I_{A}\left(G_{2}\right)-I_{A}\left(G_{1}\right))}$$

or, taking a logarithm with base 2,

(10) $$\log_{2}\left(p_{Bonferroni}\left(S,G_{1},G_{2}\right)\right)\leq I_{A}\left(G_{1}\right)+I_{A}\left(G_{2}\right)-I_{0}\left(G_{2}\right)$$

Equation (10) gives us an upper bound for the *p*-value. As expected, it depends on the lengths of bitstring encodings of *S* in $G_{1}$ and $G_{2}$ and the null hypothesis, which is only impacted by the size (number of nodes) of $G_{2}$.

The use of encoding-induced probability distribution on the power set of $G_{1}$ to generate weights for weighted Bonferroni correction is an unassuming, but significant novelty of this method. It leads to Equation (10), which brings a key innovation, as it allows for CTD to be applied to a pair of graphs, instead of using it on a singular graph. Furthermore, Equation (10) can be easily generalized to multiple graphs by repeating the same approach for correcting for multiple testing.

### 2.3. Setup for Synthetic Graph Generation

Using weighted Bonferroni correction weakens the statistical power and yields statistical significance that is lower than the statistical significance of *S* in the proposer graph. Estimating the precise impact of the Bonferroni correction on the resulting *p*-value is too computationally expensive. Therefore, an empirical approach is used. We synthesize pairs of graphs $\left(G_{1},G_{2}\right)$ of varying sizes and densities, pick different common node subsets *S*, apply the proposed method and measure the resulting *p*-value. By exploring different combinations of parameters, we are able to find empirical limits of the method and show when it can be used to yield a *p*-value that is small enough to be used for better understanding of metabolite relationships or identifying disease markers.

**Figure 2:**
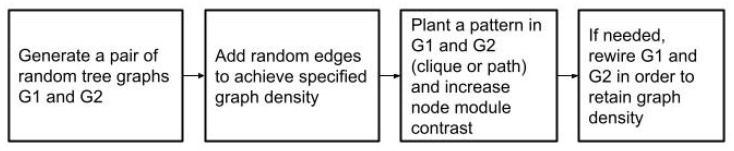
Employed synthetic graph generation method.

The graph generation procedure consists of three steps. Firstly, two random connected graphs $G_{1}$ and $G_{2}$ with the specified parameters are generated. Then, the chosen common subset of nodes *S* and the pattern *F* induced by it are planted into the graphs and the weights of edges in the planted graph are increased, which generates a contrast between the planted module and the remainder of the graph. Finally, as planting possibly added new edges to $G_{1}$ and $G_{2}$, the graphs are rewired and pruned in order to preserve the density specified by the input parameters. This workflow is depicted in Figure 2.

For the ease of verification of test results, the synthesized graphs need to be connected. Few existing random graph generators can be applied to the problem of generating random connected graphs with a planted subgraph. A majority of previous attempts repeatedly use a random graph generator to generate a graph according to the Erdos–Renyi model [12], until the generated graph ends up being connected. We chose to generate a random tree and then keep randomly adding edges until the specified density is reached, then plant *S* and *F* and possibly rewire. This approach leads to a slight bias in the distribution of graphs that are generated but benefits from a predictable execution time. Alternatively, recent development of Complex Graph Fourier Transform for surrogating graph data [13] could possibly be used to generate the synthetic graphs needed for experiments, given that controlling the second smallest eigenvalue of the graph Laplacian guarantees that the generated graph will consist of a single connected component.

In order to explore two ends of the connectedness spectrum, the planted graphs are chosen to be a path graph or a clique. It is to be expected that a clique will be discoverable with a much lower contrast than a path graph.

### 2.4. Setup for Application to Metabolomic Co-Perturbation Networks

Metabolite co-perturbation networks of diseases contain differing numbers of nodes. Even though the proposed approach expects $G_{1}$ and $G_{2}$ to have the same label sets or that there is a node correspondence between them, for the CTD encoding algorithm to work, only the nodes chosen for *S* need to necessarily exist in both networks. Therefore, a relaxed node set overlap constraint can be applied, without the need for making significant changes to the algorithm, where only node correspondence on subgraphs induced by *S* in $G_{1}$ and $G_{2}$ is required.

When working with metabolite co-perturbation networks, it is natural to choose a disease module (set of expertly curated co-perturbed metabolites important for the disease) of the proposer graph as *S*. However, some of the metabolites in the disease module of the proposer graph could be excluded from the tester graph, making the relaxed node set overlap constraint unfulfilled. To fulfill it, the problematic nodes and their incident edges can be removed from the proposer network. An alternative approach is to add the missing nodes to the tester network and leave them isolated. Both modifications possibly impact the statistical power of the method but not its correctness. For the tests conducted and presented in Section 3.3, the approach of node removal was used.

Finally, edges in metabolite co-perturbation networks can have negative weights between nodes corresponding to a substrate and a product around a perturbed enzyme [5]. However, this negative weight still expresses connectedness. Therefore, to transform the metabolite co-perturbation networks to the appropriate network model needed for applying our method, weights of all edges in the networks are substituted with their absolute value.

### 2.5. Platforms and Software Tools

All code related to this research was written in Python 3, and it is publicly available. Graph generation and manipulation were implemented using NetworkX Python package [14]. Gephi [15] was used in order to visualize the graphs and conduct data exploration for the purposes of manual checking of the method for smaller graph sizes.

The total of 664 experiments conducted during this research demanded significant computational power available only on the cloud infrastructure. The time complexity of CTD is tough to estimate precisely because of the use of a network walker that is sensitive not only to the size, but also to the topological structure of the network. However, CTD was not a limiting factor with regard to the total execution time of the experiments, completing in seconds, whereas synthetic graph generation took longer periods of time measured in hours when executing a simulation batch with higher specified network density. To facilitate faster experiment execution, we have created several Python scripts and wrapped them into command line tools using the Common Workflow Language [16]. This allows for the execution of the scripts on the Cancer Genomics Cloud platform [17]. Another advantage of such approach is simple reproducibility of the obtained results together with portability of the created tools across several different platforms enabled by Docker [18] light virtualization.

## 3. Results

The main results of this paper can be divided into three categories, each of them presented in a corresponding subsection. Firstly, we derive theoretic bounds for the minimal size of a node module discoverable by our approach of applying CTD two times and using weighted Bonferroni correction to obtain a *p*-value. Afterwards, in order to explore the impact of other parameters, such as graph density and contrast of the node module, we generate a series of synthetic graphs on which we run tests. Finally, we apply our approach to real metabolite perturbation networks for two similar metabolomic disorders, in order to check the applicability of the method.

### 3.1. Lower Bound on the Size of a Minimal Discoverable Node Subset

Very small node subsets can be hard to discover using CTD. In order to estimate the minimal size of a common node subset *S* that can be statistically significant, we can observe the best case, where the bitstring encoding of *S* contains all the nodes in *S* and no other nodes.

**Lemma** **1.** *Let $G_{1}\left(V_{1},E_{1}\right)$ and $G_{2}\left(V_{2},E_{2}\right)$ be two weighted graphs with identical node labels ($V_{1}\equiv V_{2}$) or two graphs with an established node correspondence, let V be the set of their node labels, and let |V| represent the cardinality of their node sets. Let *S* be a node subset of V that is significantly smaller than V (|S| < $\frac{2|V|}{log2(|V|)}$). Then, in the ideal case, the *p*-value of a node subset *S* in both $G_{1}$ and $G_{2}$ corrected by weighted Bonferroni correction is bounded as follows:*

(11) $$p_{Bonferroni\_ideal}\left(S,G_{1},G_{2}\right)\leq 4{\left(\frac{4}{\left|V\right|}\right)}^{|S|-2}.$$

**Proof** **of Lemma 1.** Let us run CTD on *G* and let *S* be encoded with a bitstring of length l(S) and found be the number of ones in that bitstring (the number of nodes in *S* that were successfully encoded in the bitstring). As stated in [4], length of an encoding given by CTD can be calculated as

(12) $$I_{A}\left(G\right)=\left(\left|S\right|-found+1\right)*log_{2}\left(\left|V\right|\right)+l\left(S\right)-1,$$

and, as *S* is much smaller than *V*, the encoding by the null hypothesis is given by

(13) $$I_{0}\left(G\right)=\left|S\right|*log_{2}\left(\left|V\right|\right).$$

Consider the ideal case when the bitstring encodings of *S* in both $G_{1}$ and $G_{2}$ contain all the nodes in *S* and no other nodes. Then, Equation (12) simplifies to

(14) $$I_{A}\left(G_{1}\right)=I_{A}\left(G_{2}\right)=log_{2}\left(\left|V\right|\right)+l\left(S\right)-1=log_{2}\left(\left|V\right|\right)+\left|S\right|-1.$$

According to Equation (9), we have:

(15) $$p_{Bonferroni}\left(S,G_{1},G_{2}\right)\leq 2^{-(I_{0}\left(G_{2}\right)-I_{A}\left(G_{2}\right)-I_{A}\left(G_{1}\right))}.$$

Substituting Equations (13) and (14) into Equation (15), we obtain:

(16) $$p_{Bonferroni\_ideal}\left(S,G_{1},G_{2}\right)\leq 2^{-(\left|S\right|*log_{2}\left(\left|V\right|\right)-2*\left(log_{2}\left(\left|V\right|\right)+\left|S\right|-1\right))}.$$

After simplifying Equation (16), we obtain the inequality stated by Equation (11), which we wanted to prove. □

**Lemma** **2.** *In order to obtain a statistical significance of at least $p_{wanted}$ according to the bounds given by the Algorithmic significance theorem [11], the chosen subset of nodes *S* needs to contain at least $2+\frac{2-\log_{2}\left(p_{wanted}\right)}{\log_{2}\left(\left|V\right|\right)-2}$ nodes.*

**Proof** **of Lemma 2.** Obviously, $p_{Bonferroni}$ can never be lower than in the ideal case described in the proof of Lemma 1. Therefore, if the *p*-value for this ideal case is larger than some threshold $p_{wanted}$, the *p*-value for the other cases can not be lower than $p_{wanted}$. In order to ensure that, in the ideal case, we can achieve a wanted level of statistical significance, we strictly enforce $p_{Bonferroni\_ideal}$ to be smaller than $p_{wanted}$ by enforcing the upper bound on $p_{Bonferroni\_ideal}$ to be bounded by $p_{wanted}$.

The aforementioned bound enforcement can be stated as

(17) $$p_{Bonferroni\_ideal}\left(S,G_{1},G_{2}\right)\leq 4{\left(\frac{4}{\left|V\right|}\right)}^{|S|-2}\leq p_{wanted}.$$

Taking a logarithm with base 2 and solving for |S| directly leads to

(18) $$\left|S\right|\geq 2+\frac{2-\log_{2}\left(p_{wanted}\right)}{\log_{2}\left(\left|V\right|\right)-2},$$

which is the statement we wanted to prove. □

Note that Lemma 2 does not state that by choosing a node subset larger than some threshold value we can ensure that the *p*-value will be smaller than $p_{wanted}$; it only establishes a bound on when the algorithmic significance theorem can be applied to estimate statistical significance. In other words, it is possible that a smaller subset of nodes could yield a higher connectedness, but its statistical significance could not be proven by our approach.

An important consequence of Lemma 2 is that we can skip tests on some parts of parameter space for graph generation, as we already know that for that part of the parameter space we can not get statistical significance, speeding up the parameter space exploration. For example, when synthesizing graphs with 1000 nodes, a minimal discoverable node subset with a statistical significance of 0.05 contains at least three nodes. However, if we change the wanted statistical significance threshold to $5\times 10^{-10}$, the size of the minimal discoverable node subset increases to 7, even though we changed the statistical significance threshold by 8 orders of magnitude. This slow increase shows that a minimal size of the shared node module is not a significant limiting factor on the applicability of the method. This is why, in the next subsection, we employ synthetic graph generation in order to test the impact of other parameters that are harder to constrain theoretically.

### 3.2. Empirical Results on Synthetic Graphs

In order to see how the effectiveness of the proposed approach is impacted by other graph and node module features, we have generated random synthetic graphs defined by five parameters. The values of parameters were assigned to be similar to potential biological use cases—disease-specific metabolite co-perturbation networks contain between 300 and 1000 nodes [4,5], while gene co-expression networks consist of thousands of nodes [19], but the connectedness patterns remain significantly smaller, consisting of no more than 1% of network nodes. An overview of the parameters used for synthetic graph generation and their values is given in Table 1.

Average number of neighbours for each node (average unweighted node degree) is a more descriptive parameter than graph density if the number of nodes in the graph is also known and is therefore chosen instead of graph density. A higher number of neighbours makes it harder for CTD to detect a pattern as statistically significant because the probability diffusion scheme used for encoding by the CTD is based on recursively distributing probability to neighbouring nodes. Therefore, a larger neighbourhood leads to larger probability dispersion and a weaker signal.

The impact of the percentage of nodes in *G* that are included in *S* has already been discussed in Section 3.1. and has been shown to not be a significant limiting factor to the applicability of the method, especially if a relatively weak measure is used to determine if a result is statistically significant, such as requiring a *p*-value to be lower than 0.05 as is often the case in medicine or biology.

As mentioned in Section 2.3, node module contrast is defined as a relative difference in the average edge weight in the planted node module and in the remainder of the graph. It is important to note that the subgraphs induced by *S* in *G*_1_ and *G*_2_ could contain edges that are not in the planted node module contained in *S*. That is why node module contrast specifically takes into consideration only the edges in *S* that belong to the planted module.

The type of the planted pattern (a path graph or a clique) will influence the ability of the method to detect the pattern. A path maximizes the dispersion of probability to the remainder of the graph, while a clique minimizes it. Therefore, these two patterns define the best and worst case for the CTD algorithm in the spectrum of Hamiltonian graphs.

**Figure 3:**
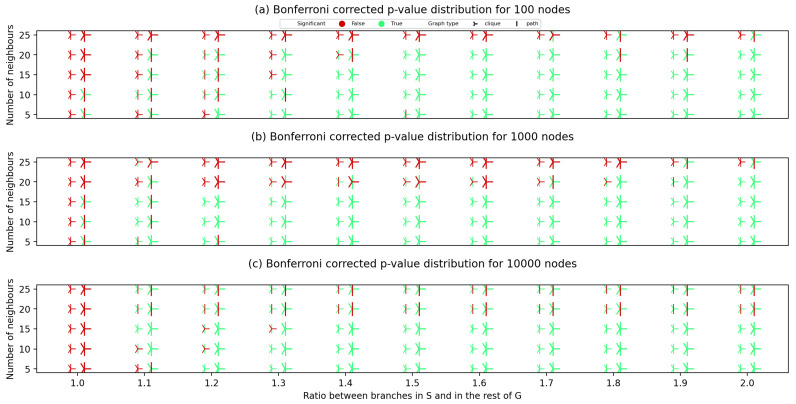
The distribution of Bonferroni-corrected *p*-values for different randomly generated synthetic graphs with 100 (**a**), 1000 (**b**) and 10,000 (**c**) nodes. The *x*-axis shows the node module contrast (the ratio between branch weights in the planted *S* module and in the rest of the graph). The *y*-axis displays the average number of neighbouring nodes for each node (average unweighted node degree). Smaller marker relates to the S node set with five nodes in (**a**,**b**) and 50 nodes in (**c**), while the larger stands for *S* with 10 nodes in (**a**,**b**) and 100 nodes in (**c**). “|” marker corresponds to the case when a path graph is planted, while “Y” corresponds to the case where a clique is planted via *S*. A *p*-value is considered significant if it is lower than 0.05.

After creating the synthetic graph pairs, for each created graph pair, we have calculated the Bonferroni corrected *p*-value. The results of applying our approach to the synthetically generated graphs are given in Figure 3, which represent the results on graph pairs with 100, 1000 and 10,000 nodes.

Comparing diagrams in Figure 3, we can conclude that all the hypotheses on the impact of parameters on the obtained statistical significance were correct. Graphs with larger density (higher average number of neighbours) suffer more from dispersed probability and are more likely to lead to the algorithm yielding an unsatisfactory *p*-value. Graphs pairs that had a clique planted obtain a satisfactory statistical significance more commonly compared to the ones where a path graph was planted. A significant impact of node module contrast can be seen by constructing imaginary hypercurves that would separate the statistically significant points (green on the figures) from the ones with a *p*-value larger than 0.05. A majority of the graphs with 15 and less neighbouring nodes with node module contrast higher than 1.2 also obtain a statistical significance when searching for *S* in $G_{2}$.

### 3.3. Application on Metabolite Perturbation Networks

In order to test the applicability of our approach on metabolite perturbation networks, two metabolic disorders argininemia (ARG) and Rhizomelic chondrodysplasia punctata (RCDP) were chosen, based on a high similarity between their disease modules, as discussed in [5]. Metabolite perturbation networks for ARG and RCDP contain 430 and 381 nodes, respectively, making them comparable in size with the synthesized networks discussed in Section 3.2. *S* is chosen as the known disease module for ARG and contains 22 nodes. However, the networks need to be preprocessed and adjusted to apply our approach. The methodology behind these adjustments is discussed in detail in Section 2.4.

Table 2 shows results of tests on ARG and RCDP metabolite co-perturbation networks. The first row represents the results of applying the approach on the original ARG and RCDP networks. The following rows correspond to the tests where a clique with increased contrast was planted inside the node module of the tester graph (in this case the RCDP network).

As shown in Table 2, the node module contrast in $G_{2}$ is lower than 1, meaning that the node module is less connected than the rest of $G_{2}$. The contrast in $G_{1}$ is not as important, as *S* was chosen as the known disease module for ARG; therefore, its occurrence probability in $G_{1}$ according to the CTD induced probability distribution is guaranteed to be high. Looking at Figure 3, it is expected that no statistical significance would be observed for graphs of this size and node module contrast in the tester graph, and that is exactly the case.

As the node module contrast in $G_{2}$ is low, the node module is situated in a sparser part of the network. Upon further inspection, the *S*-induced subgraph in the RCDP network is not connected. Therefore, even if the contrast was higher, the probability diffusion walker would need to incur misses, venturing outside of *S* in order to encode the nodes from two unconnected parts of the *S*-induced subgraph. This means that the choice of *S* as the disease module of Argininemia is ill-suited for being the shared highly connected node module between ARG and RCDP, as seen from the fact that our approach detected no statistical significance (row 1 of Table 2).

One might wonder if a slight change in structure of the *S*-induced subgraph or a choice of different *S* with higher contrast in $G_{2}$ would lead to statistical significance. As the weighted Bonferroni correction based on the probability distribution in $G_{1}$ already accounts for the choice of *S*, we could possibly have chosen another *S* which would be closer to the ideal case, without the need to further correct for multiple testing. Therefore, the remaining tests are run with a slightly modified $G_{2}$, in which a clique was planted inside the node module. Additionally, node module contrast of the planted clique was progressively increased. The results of tests on the modified graphs correspond to rows 2–4 of Table 2, and they clearly demonstrate that better connectedness and contrast are needed for the pattern to be detected.

These results show that node module contrast can impact the boundary for *p*-value by orders of magnitude, as higher contrast yields less probability dispersion to nodes outside of *S*, which in turn lead to a lower bitstring encoding length. Additionally, the importance of choosing *S* is demonstrated, as is the sensitivity of the algorithm to the connectedness expressed by *S*.

## 4. Discussion

Our work establishes information-theoretic upper bounds on the *p*-values for localized pattern of similarity between two labeled weighted graphs, where the similarity consists of a set *S* of nodes that are identically labeled and highly connected in both graphs. Our results are independent of the algorithm used to detect *S* and thus pave the way toward future practical algorithmic implementations.

Our work extends the recently proposed CTD method [4]. From the definition of the CTD method and the derived theoretical work shown in Section 2, the choice of the encoding scheme will impact the *p*-value bound, but not the correctness of our approach. Therefore, modifications of the encoding scheme are also potentially fruitful directions of future work, as they would allow for discovery of a broader scope of connectivity patterns induced by shared highly connected node modules. These modifications could include tuning of parameters of the diffusion algorithm, such as the stopping threshold or complete replacement of the diffusion scheme with, for example, a slightly modified graph search algorithm such as $A^{*}$ [20].

For the ease of understanding, the proposed method requires node sets of $G_{1}$ and $G_{2}$ to be equal or a node correspondence to exist between the graphs. However, as briefly discussed in Section 2.4 and Section 3.3, even though at first glance this looks like a significant limitation, it can be easily circumvented by extending the node sets of graphs to include all the nodes from the other graph’s node set, but leaving them isolated. This is a valid approach, as it can only weaken the signal but never lead to false deductions. Especially in the field of metabolomics, the metabolite co-perturbation networks are constructed from samples that always measure the same set of metabolites, but some of the metabolites are pruned from the specific disease’s perturbation network as they are deemed unimportant for the disease and only cause noise. However, if the pruning step is omitted or reversed, the metabolite co-perturbation networks can easily be equalized to the same (starting) set of node labels.

Alternatively, a relaxed criterion can be applied, only requiring that both graphs contain all nodes in the node module. This is valid because the size of $G_{1}$ is implicitly accounted for by the constructed probability distribution used to penalize the weighted Bonferroni correction, and the size of $G_{2}$ is taken into account by the CTD algorithm when calculating the *p*-value of *S* in $G_{2}$. Therefore, if there are some nodes in the node module that do not exist in one of the graphs, the same approach of adding them as isolated nodes can be applied.

A third way of satisfying the criteria for applying our method is removing the problematic nodes from the node module altogether. This approach is easiest to implement and was used on the real world example presented in Section 3.3. However, this method is ill suited for situations where a large number of nodes would need to be removed, as each removed node increases the probability of removal of a node on the Hamiltonian path in the node module. That would lead to drastic degradation of either the *p*-value of the node module in the tester graph or the probability of node module in the proposer graph, ultimately having a devastating impact on the Bonferroni corrected *p*-value and possibly discarding the shared connectedness pattern expressed by the node module. This is a possible explanation of the problems that the method was experiencing on the presented real world example before planting of the connectedness pattern.

By using a weighted Bonferroni correction, our approach already accounts for multiple testing on the power set of $G_{1}$; therefore, one could consider all subgraphs of $G_{1}$ and calculate the Bonferroni corrected *p*-value of their corresponding node modules for the graph pair $\left(G_{1},G_{2}\right)$ without the need to apply any further corrections. As the cardinality of a power set is exponentially related to the size of $G_{1}$, the complexity of such approach would be computationally impossible for any real applications. Therefore, development of a heuristic method of choosing the node module *S* is a natural direction of future work to be explored.

## Figures and Tables

**Table 1:** List of parameters used for generating pairs of random synthetic graphs.

Graph Parameter	Set of Values
Number of nodes in graphs *G*_1_ and *G*_2_	100, 1000, 10,000
Average number of neighbours for each node	5, 10, 15, 20, 25
Percentage of graph nodes that are in *S*	0.5%, 1%
Node module contrast	1.0, 1.1, 1.2, 1.3, 1.4, 1.5, 1.6, 1.7, 1.8, 1.9, 2.0
Type of the graph planted by *S*	Path, Clique

**Table 2:** Results of applying our approach to Argininemia (ARG) and Rhizomelic chondrodysplasia punctata (RCDP) metabolite co-perturbation networks. ARG network takes place of the proposer graph $G_{1}$ and the RCDP network is used as the tester graph $G_{2}$. Node module contrast is defined as the relative difference in average edge weight in the node module and in the remainder of the graph.

Test Case	Planted Module	Node Module Contrast in $G_{2}$	Upper Bound for *p*-Value
Original ARG and RCPD networks	None	0.72	$8.06\times 10^{23}$
Planted graph with original contrast	Clique	0.72	$4.24\times 10^{23}$
Planted graph with ×2 original contrast	Clique	1.44	$5.73\times 10^{17}$
Planted graph with ×3 original contrast	Clique	2.16	$2.01\times 10^{-31}$

## Data Availability

The data and the source code to reproduce experiments conducted in this research are available in public repository https://github.com/predragoetf/CTD2 accessed on 20 September 2022.
